# *DHD6* Delays Flowering in Rice by Negatively Regulating the Expression of *Ehd1*

**DOI:** 10.3390/plants14223503

**Published:** 2025-11-17

**Authors:** Qiping Sun, Le Song, Juan Zhao, Jinxia Yun, Zhenhua Guo, Gan Sha, Lei Yang, Renjian Li, Rashmi Jain, Artur Teixeira de Araujo Junior, Zihao He, Yin Wang, Qun Yang, Jiandi Xu, Xia Li, Pamela C. Ronald, Guotian Li

**Affiliations:** 1 State Key Laboratory of Agricultural Microbiology, Hubei Hongshan Laboratory, Hubei Key Laboratory of Plant Pathology, The Center of Crop Nanobiotechnology, Huazhong Agricultural University, Wuhan 430070, China; 15610372270@163.com (Q.S.); lesong0819@163.com (L.S.); zhaojuan201809@163.com (J.Z.); scienceshagan@163.com (G.S.); ylstudy@webmail.hzau.edu.cn (L.Y.); lirenjian@webmail.hzau.edu.cn (R.L.); wangyin02021@163.com (Y.W.);; 2 College of Chemistry and Life Sciences, Sichuan Provincial Key Laboratory for Development and Utilization of Characteristic Horticultural Biological Resources, Chengdu Normal University, Chengdu 611130, China; 3 National Key Laboratory of Crop Genetic Improvement, Huazhong Agricultural University, Wuhan 430070, China; yunjx@lzu.edu.cn (J.Y.);; 4 MOE Key Laboratory of Cell Activities and Stress Adaptations, School of Life Sciences, Lanzhou University, Lanzhou 730000, China; 5 National Key Laboratory of Green Pesticide, Guangdong Province Key Laboratory of Microbial Signals and Disease Control, South China Agricultural University, Guangzhou 510642, China; 6 Department of Plant Pathology and the Genome Center, University of California, Davis, CA 95616, USAarturtaj@gmail.com (A.T.d.A.J.); 7 Institute of Wetland Agriculture and Ecology, Shandong Academy of Agricultural Sciences, Jinan 250100, China; 8 The Joint BioEnergy Institute, Emeryville, CA 94608, USA; 9 Innovative Genomics Institute, University of California, Berkeley, CA 94720, USA

**Keywords:** heading date, rice, *Delayed Heading Date 6*, *Ehd1*

## Abstract

Heading date, also known as flowering time, plays a crucial role in determining the regional adaptability of rice (*Oryza sativa* L.). Heading date is regulated by numerous genes involved in various photoperiod pathways. Here, we isolated the *Delayed Heading Date 6* (*DHD6*) gene from a whole-genome-sequenced rice mutant population. We demonstrated that a 2 bp deletion in the coding region of *DHD6* truncates the protein and confers early flowering. Genetic analysis shows that *DHD6* functions upstream of *Ehd1* and is synergistic with *Se14* and *PHYC* to regulate flowering time. In addition, we identified natural alleles of *DHD6* that are associated with heading date and likely contribute to the geographic adaptation of rice. In summary, DHD6 functions upstream of *Ehd1*, reducing the transcriptional level of *Ehd1*, thereby delaying flowering.

## 1. Introduction

Rice (*Oryza sativa*) is a staple crop that feeds over half of the world’s population, playing a key role in global food security [1,2]. The heading date in rice, commonly referred to as flowering time, is an agronomic trait crucial to local adaptation and yield potential of rice, which has been extensively studied [3]. In rice, a typical short-day crop, photoperiod sensitivity determines the critical day length required to trigger phase transitions through interactions with the circadian clock. The photoperiod-responsive florigens Hd3a (Heading date 3a) and RFT1 (RICE FLOWERING LOCUS T1), induced under short-day (SD) and long-day (LD) conditions, respectively, are synthesized in the leaf phloem and translocated to the shoot apical meristem to initiate flowering [4,5]. These proteins act as mobile signals for the induction of flowering.

There are two major photoperiod-related pathways regulating heading date in rice. Under SD conditions when day length is less than 13.5 h [6], the OsGI-Hd1-Hd3a (OsGIGANTEA-Heading date1-Hd3a) pathway plays a major role [7], and increased expression of Hd3a induces flowering. Under LD conditions, heading date is primarily regulated by the Ghd7-Ehd1-Hd3a/RFT1 (Grain number, plant height and heading date 7-Early heading date 1-Hd3a/RFT1) pathway, which is unique to rice [7]. There is an interconnection between these two regulatory pathways of flowering time. Recent studies have revealed that Hd1 forms a repressive complex with Ghd7 and/or DTH8 (Days-to-heading on chromosome 8) to regulate heading date [8]. Besides these core regulators, other regulators include phytochrome genes PHYTOCHROME A (PHYA), PHYB, PHYC, and several epigenetic factors also participates in flowering regulation [9,10,11]. Histone H3 lysine 4 methylation (H3K4me) plays a key role in regulating flowering time by modifying chromatin structure in the promoter regions of related genes [9]. Se14, encoding a JmjC domain-containing protein, suppresses rice flowering through demethylation of H3K4me3 at the RFT1 locus [12].

*Ehd1* encodes a B-type response regulator and promotes the expression of *Hd3a* and *RFT1* under both SD and LD conditions [13,14]. Ehd1 is thus the central hub in the regulatory network of rice flowering, and its expression is regulated by many proteins. Ehd2, Ehd3, Ehd4, Ehd5, OsMADS50, OsMADS56, OsTrx1 (*Oryza sativa* Trithorax1), and SIP1 (SDG723/OsTrx1/OsSET33 INTERACTION PROTEIN 1)/OsSUF4 positively regulate *Ehd1* expression [15,16,17,18,19,20,21]. In contrast, bZIP71, DHD1 (Delayed Heading Date1), Ghd7, Ghd8, HBF1, OsGI, and OsRE1 negatively regulate *Ehd1* expression [22,23,24,25,26,27,28]. SIP1, HBF1, and OsRE1 are transcription factors that directly bind to the promoter of *Ehd1* [17,24,25].

The WD40 repeat proteins, which are highly conserved across eukaryotes, play crucial roles in multiple signaling pathways [29]. *Ehd5* encodes a WD40 domain-containing protein that promotes flowering by regulating *Ehd1* and *Hd3a* expression through interactions with Roc4 (Rice outermost cell-specific gene 4) and Ghd8 [21]. OsWDR5a (WD repeat domain 5a) interacts with OsTrx1 and promotes flowering and panicle branching in rice by regulating H3K4me3 levels at the *Ehd1* locus [30]. For other traits, *OsKRN2* in rice encodes a WD40 protein that cooperates with DUF1644 to regulate grain number in rice [31]. There are still many WD40 repeat domain-containing proteins that need further investigation.

Here, we identified an early flowering mutant FN75 by screening a whole-genome-sequenced, fast-neutron–induced mutant population in the Kitaake genetic background [32,33,34]. The causative gene *Delayed Heading Date 6* (*DHD6*) was identified using whole-genome sequencing data combined with co-segregation analyses. *DHD6* is mutated by a 2 bp deletion. *DHD6* encodes a WD40 domain-containing protein that reduces *Ehd1* expression to inhibit flowering. Genetic studies demonstrate that the *dhd6 ehd1* mutant has the same heading date as the *ehd1* line, indicating that *DHD6* regulates heading date via *Ehd1*. The *dhd6 se14 phyC* triple mutant shows the earliest heading date, indicating the synergistic function of these negative regulators of flowering time in rice. Analysis of 3K-sequenced rice varieties revealed that natural *DHD6* alleles are associated with heading date variation, suggesting their potential role in regional adaptation of cultivated rice.

## 2. Results

### 2.1. Identification of an Early Flowering Mutagenized Line FN75

**Figure 1:**
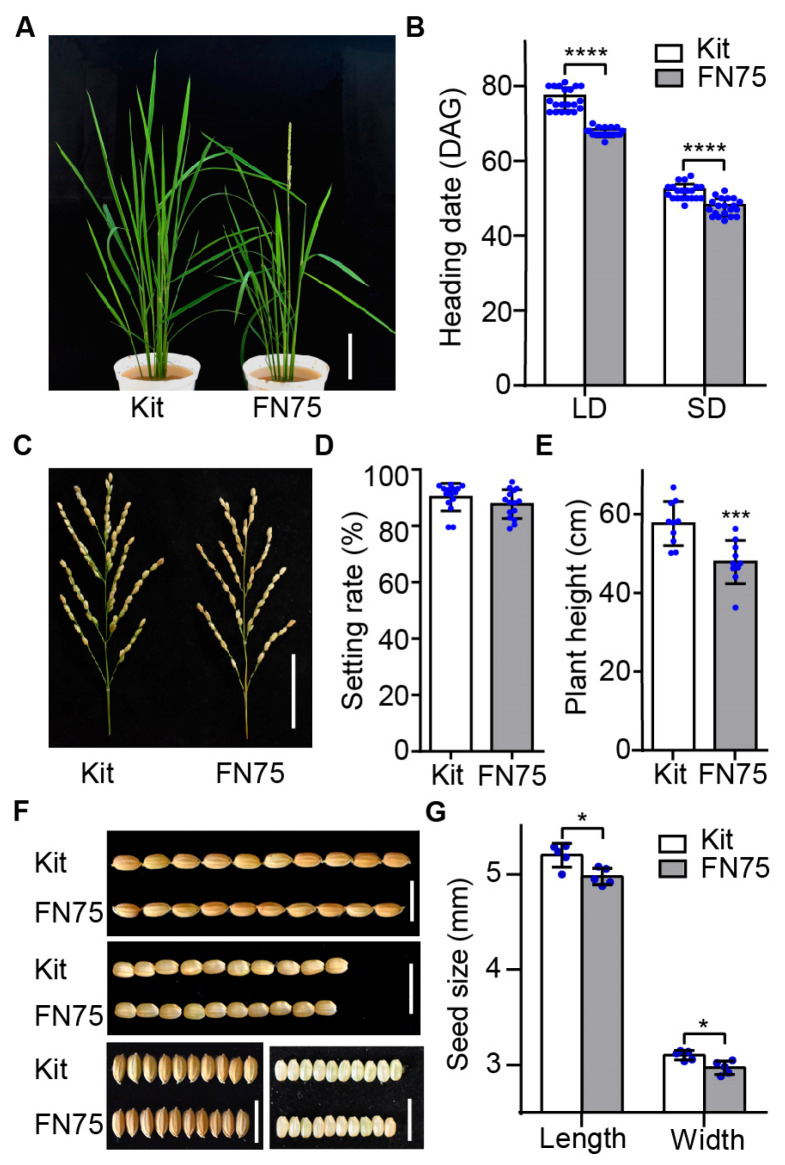
The early flowering line FN75. (**A**) Line FN75 and the control line Kitaake (Kit) at the heading stage grown under natural long-day (LD) conditions. Bar, 10 cm. (**B**) Heading dates of Kitaake and FN75 under LD (in Wuhan) and natural short-day (SD) conditions (in Hainan) during winter. The heading date was scored from germination to the emergence of the panicle (heading day) from the main culm (*n* = 20). DAG, days after germination. (**C**) Major panicles of Kitaake and FN75. Bar, 5 cm. (**D**) Seed setting rates of Kitaake and FN75 (*n* = 15). (**E**) Plant height of Kitaake and FN75 (*n* = 10). (**F**) Grains of Kitaake and FN75. Bars, 1 cm. (**G**) Grain length and grain width of Kitaake and FN75 (*n* = 5). Data are shown as the mean ± SD, and asterisks indicate significant differences using the unpaired Student’s *t*-test (* *p* < 0.05, *** *p* < 0.001, **** *p* < 0.0001).

Kitaake, an early flowering rice variety, has been used to accelerate rice genetic studies [32]. To identify lines with even earlier flowering, we visually screened over 1500 fast-neutron (FN)–induced mutant lines of KitaakeX, a transgenic Kitaake line expressing the Xa21 immune receptor that flowers concurrently with Kitaake [34]. From the screen, we identified several lines that flowered earlier than KitaakeX, among which FN75 was of particular interest due to its lack of obvious growth defects (Figure 1A). Additionally, from the whole-genome sequencing data, the identified mutations were not in genes known for the regulation of heading date (Table S1), suggesting that a previously uncharacterized gene is responsible for the early flowering phenotype. After crossing FN75 with Kitaake, FN75 lines lacking the *Xa21* gene were selected from the F2 segregating population for subsequent studies on heading date. We analyzed the heading date of the FN75 line under both long-day (LD) and short-day (SD) conditions. Under LD conditions (30°28′ N), the heading date of FN75 is 54.8 days after germination, which is 3.8 days earlier than its parental line Kitaake. Under SD conditions (18°23′ N), the FN75 line flowers 4.5 days earlier than Kitaake (Figure 1B). The plant height of FN75 is 17% shorter than that of Kitaake (Figure 1E). Compared with the wild-type, the pollen fertility and the seed setting show no difference; the grain size is slightly reduced (grain length, 7.6 vs. 7.4 mm; grain width, 3.7 vs. 3.5 mm) (Figure 1C,D,F,G). These results demonstrate that the rice mutant line FN75 flowers earlier than Kitaake and shows normal seed setting.

### 2.2. Cloning of the DHD6 Gene Using Whole-Genome Sequencing

**Figure 2:**
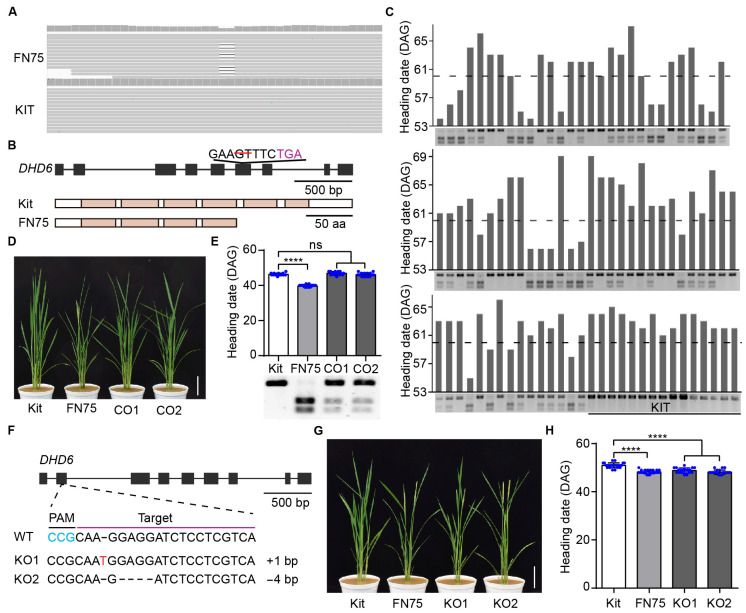
The 2 bp deletion in gene *DHD6* causes early flowering. (**A**) Integrative Genomics Viewer (IGV) snapshot of the 2 bp deletion in the sequenced heterozygous FN75 line. Sequence reads aligned to the KitaakeX (KIT) reference genome are marked in gray. (**B**) Gene structure of *DHD6* in line FN75. The red line represents the deletion. The stop codon is marked purple. Gray boxes indicate exons and lines for introns. In protein structures, brown boxes indicate WD domains; aa, amino acid. (**C**) The 2 bp deletion on chromosome 5 of line FN75 co-segregates with the early flowering phenotype in the segregating F2 population. DAG, days after germination. Cleaved Amplified Polymorphic Sequence (CAPS) markers were used in genotyping. A large band indicates the presence of at least one parental allele in the plant. (**D**) Kitaake (Kit), FN75, and the complementation lines CO1 and CO2 at the heading stage. Bar, 10 cm. (**E**) Heading dates of Kitaake, FN75, CO1, and CO2 under natural long-day (LD) conditions (*n* = 15). For genotyping using the CAPS marker, three bands indicate the complementation lines. (**F**) Gene editing of *DHD6*, the sequence of the guide RNA (in purple) with the PAM sequence in blue. Gray boxes indicate exons and lines for introns. A 1 bp insertion and 4 bp deletion in the first exon of *DHD6* were detected in the two independently edited lines KO1 and KO2. (**G**) Kitaake, FN75, KO1, and KO2 lines at the heading stage. Bar, 10 cm. (**H**) Heading dates of Kitaake, FN75, KO1, and KO2 lines under natural long-day (LD) conditions (*n* ≥ 18). Data are shown as the mean ± SD, and asterisks indicate significant differences using Tukey’s multiple comparisons test (**** *p* < 0.0001).

We employed a whole-genome sequencing approach to isolate the gene in line FN75 that is responsible for the early flowering phenotype [34]. M3 progeny derived from the sequenced FN75 line segregates for the short heading date, indicating that the parent line FN75 is heterozygous for the mutation. In the M3 segregating population, we observed 14 early flowering plants and 46 plants with a normal heading date. The χ^2^ analysis revealed that the observed value is statistically similar to the expected value at 1:3 (*p* value = 0.831), indicating that the early flowering phenotype is controlled by a single recessive mutation. We identified 40 mutations in line FN75, including 20 single-base substitutions, 17 deletions, one insertion, and two inversions, affecting eight genes in total (Table S1). To rapidly identify the causative mutation for the early flowering phenotype in line FN75, we prioritized loss-of-function mutations affecting proteins with notable functional domains. In this way, we prioritized a 2 bp deletion on chromosome 5 (Chr05: 26,962,970–26,965,906) that causes a frameshift mutation in the fourth exon of the gene *DHD6* (LOC_Os05g46570), which encodes a protein containing a cluster of six WD40 domains (Figure 2A,B). Using a segregating population of 74 plants, we observed that the 2 bp deletion on chromosome 5 co-segregated well with the early flowering phenotype (Figure 2C). The 2 bp deletion results in a premature stop codon, leading to the truncation of two out of six WD40 domains at the C-terminus of the highly conserved DHD6 protein (Figure 2B and Figure S1).

To test whether the 2 bp deletion in *DHD6* is responsible for the early flowering phenotypes in the FN75 line, we performed a genetic complementation assay. We transformed the FN75 line with the full-length *DHD6* gene, including its 1.6 kb native promoter, using *Agrobacterium*-mediated transformation and obtained two independently transformed lines. In the T1 plants, both complementation lines flowered at a similar time to Kitaake (Figure 2D,E). These results demonstrate that the 2 bp deletion in gene *DHD6* causes early flowering in line FN75.

### 2.3. DHD6 Represses Flowering in Rice

To determine the role of *DHD6* in flowering, we used CRISPR-Cas9 technology to edit *DHD6* by targeting sites near the start codon ATG and obtained 18 independent edited lines. We further characterized genome-edited lines KO1 and KO2 which carry a 1 bp insertion and 4 bp deletion, respectively, that completely disrupts the *DHD6* gene (Figure 2F). Under LD conditions, the T2 generation lines KO1 and KO2 flowered 48.8 and 48.1 days after germination, respectively, which were 2.1 and 2.8 days earlier than the parental line Kitaake, and similar to FN75 (Figure 2G,H).

**Figure 3:**
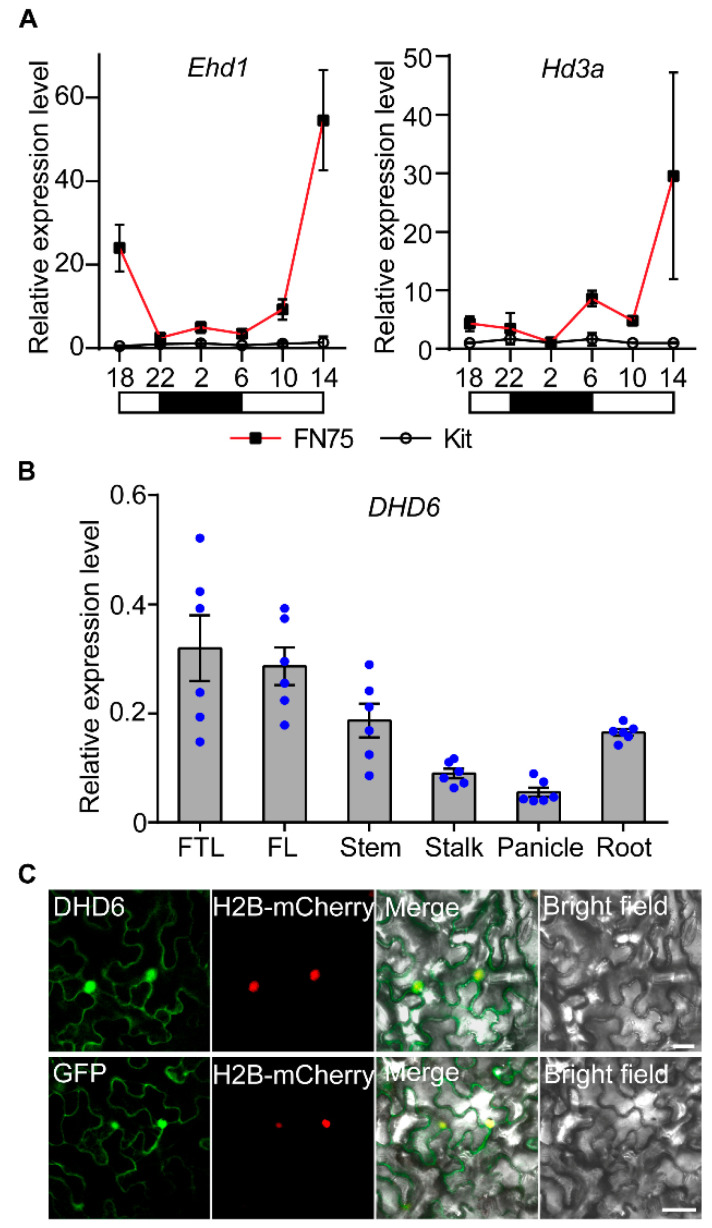
Expression assays of *DHD6* and other heading date-related genes in Kitaake (Kit) and FN75. (**A**) qRT-PCR assays of genes regulating heading date. Total RNA was extracted from leaves of 28-day-old Kitaake and FN75 plants grown under LD conditions (*n* = 3). *OsActin* was used as the internal control. Data are shown as the mean ± SD. (**B**) Relative expression of *DHD6* in different rice tissues assayed using qRT-PCR (*n* = 6). FTL, first top leaf; FL, flag leaf. *OsActin* was used as the internal control. (**C**) Subcellular localization of the DHD6-GFP fusion protein in *Nicotiana benthamiana* leaf epidermal cells, which were co-transformed with the nuclear localization marker H2B-mCherry. Bars, 10 μm.

To investigate the role of *DHD6* in the regulatory network of heading date, we analyzed the expression levels of heading date-related genes in Kitaake and FN75. Leaves of 28-day-old rice seedlings were sampled in the early morning, when *Ehd1* shows the highest expression level [13]. qRT-PCR analysis revealed that the transcriptional levels of *Ehd1*, *Hd3a*, *RFT1*, *SIP1*, and *OsMADS50*, but not *OsTrx1*, are significantly higher in line FN75 than in Kitaake (Figure S2). We then compared the circadian expression levels of *Ehd1* and *Hd3a*, which were higher in line FN75 than in Kitaake (Figure 3A). These results suggest that *DHD6* is involved in the *Ehd1*-*Hd3a*/*RFT1* pathway that regulates heading date in rice.

### 2.4. DHD6 Is Ubiquitously Expressed and Localized in the Nucleus and Cytoplasm

To examine the tissue expression pattern of *DHD6*, we used qRT-PCR assays to analyze the expression of *DHD6* in different rice tissues, including the first top leaf, flag leaf, stem, stalk, panicle, and root. *DHD6* is expressed in all the tissues with the highest level in the first top leaf (Figure 3B). When transiently expressed in *Nicotiana benthamiana*, the DHD6-GFP fusion protein was localized in both the nucleus and cytoplasm (Figure 3C). The same results were observed in rice protoplasts (Figure S3).

### 2.5. DHD6 Acts Upstream of EHd1 to Negatively Regulate Flowering Time

**Figure 4:**
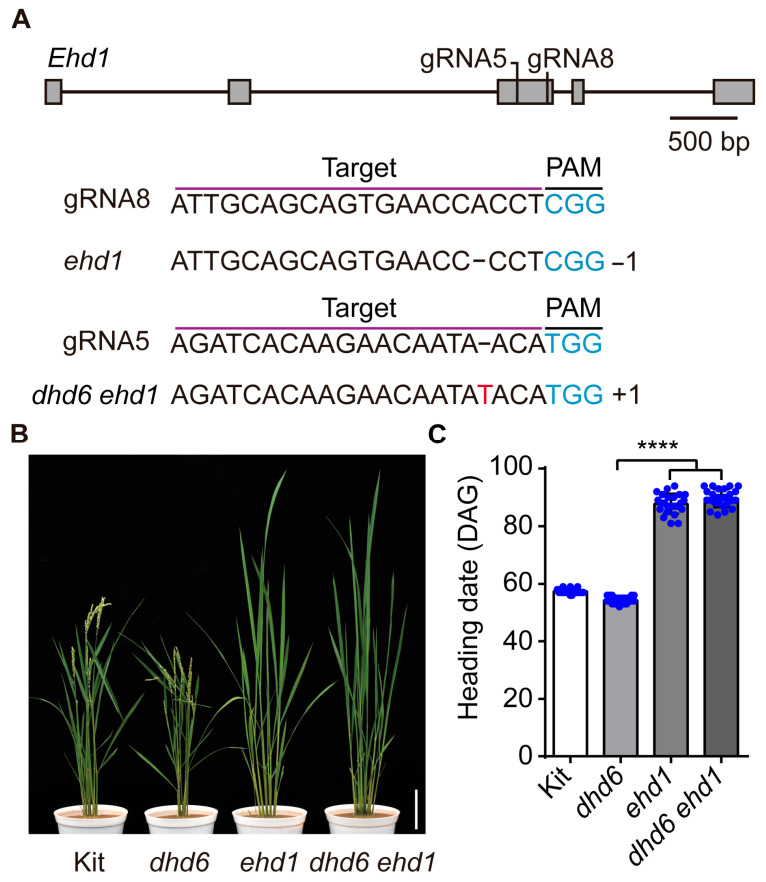
*DHD6* functions upstream of *Ehd1*. (**A**) Gene editing of *Ehd1*, the sequence of the guide RNA (in purple) with the PAM sequence in blue. Gray boxes indicate exons, and lines for introns. A 1 bp deletion and 1 bp insertion in the third exon of *Ehd1* were detected in the lines *ehd1* and *dhd6 ehd1* (gene editing of *Ehd1* in FN75). (**B**) Kitaake, *dhd6*, *ehd1,* and *dhd6 ehd1* lines at the heading stage. Bar, 10 cm. (**C**) Heading dates of Kitaake, *dhd6*, *ehd1,* and *dhd6 ehd1* lines under natural long-day (LD) conditions (*n* = 24). Data are shown as the mean ± SD, and asterisks indicate significant differences using Tukey’s multiple comparisons test (**** *p* < 0.0001).

To further investigate whether DHD6 regulates flowering through *Ehd1*, we performed gene editing of *Ehd1* in both Kitaake and FN75 lines. After PCR amplification and sequencing analysis to determine the genotypes of gene edits, we selected the *ehd1* mutant line with a 1 bp deletion in the third exon and the *dhd6 ehd1* double mutant line with a 1 bp insertion in the same exon for further investigation (Figure 4A). In the T1 generation, the heading date of the *dhd6 ehd1* double mutant was similar to that of the *ehd1* mutant, and the *dhd6 ehd1* double mutant and the *ehd1* mutant delayed flowering by 32.42 and 30.46 days, respectively, compared to Kitaake (Figure 4B,C). These results indicated that DHD6 negatively regulates the heading date by suppressing the expression of *Ehd1* in rice.

### 2.6. DHD6, Se14, and PHYC Synergistically Regulate Flowering

**Figure 5:**
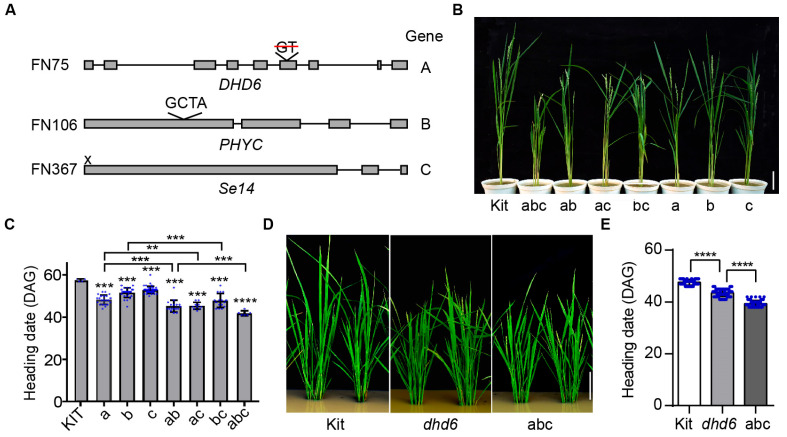
Genetic interactions of *DHD6* with genes *PHYC* and *Se14* in regulating flowering time. (**A**) Gene structures and mutation sites of *DHD6* (A), *PHYC* (B), and *Se14* (C) in lines FN75, FN106, and FN367, respectively. (**B**) Kitaake (Kit), FN75 (a), FN106 (b), FN367 (c), double mutants *dhd6 phyC* (ab)*, dhd6 se14* (ac)*, phyC se14* (bc), and the triple mutant (abc) plants at 60 days post-germination. Bar, 10 cm. (**C**) Heading dates of Kitaake, a, b, c, and derived double and triple mutants under LD conditions. DAG, days after germination. (**D**) Kitaake, *dhd6*, and the *dhd6 phyC se14* triple mutant line abc at the heading stage in the field. Bar, 10 cm. (**E**) Heading date of Kitaake, *dhd6*, and the *dhd6 phyC se14* triple mutant line abc (*n* > 50). Data are shown as the mean ± SD, and asterisks indicate significant differences using the unpaired Student’s *t*-test (** *p* < 0.01, *** *p* < 0.001, **** *p* < 0.0001).

To further investigate the genetic relationship between DHD6 and other regulators of flowering time in rice, we searched the whole-genome-sequenced mutant population and identified lines with mutations in genes known for regulating flowering time. In this approach, we identified lines FN106 and FN367, which are mutated in genes *Se14* and *PHYC*, respectively (Figure 5A). Through crossings, we generated homozygous single, double, and triple mutant lines. Under LD conditions, all the single mutant lines flowered earlier than Kitaake, with line FN75 flowering the earliest. All of the double mutant lines flowered earlier than any of the single mutant lines, and the triple mutant flowered the earliest of all these lines (Figure 5C). Notably, the triple mutant line *dhd6 phyC se14* flowered 40 days post-germination (Figure 5C–E), with mature seeds produced 60 days post-germination (Figure 5B). We grew three generations of the triple mutant in a seed-to-seed manner within 180 days in a greenhouse in Davis, California, from March to September 2018, indicating that researchers can grow the *dhd6 phyC se14* line for six generations within a year in a controlled environment. Based on these findings, we demonstrate that *DHD6*, *Se14*, and *PHYC* synergistically and negatively regulate flowering time of rice, and that simultaneous mutation of the three genes generates rice lines with a remarkably short life cycle of only two months.

### 2.7. Natural Alleles of DHD6 Are Associated with Heading Date

**Figure 6:**
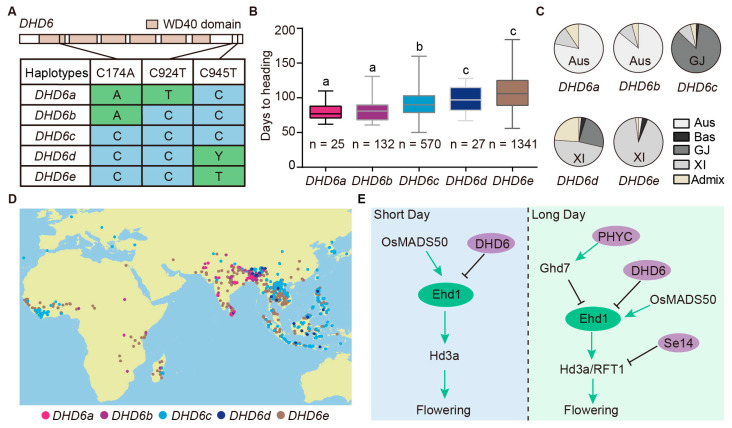
Natural variation in DHD6 is associated with the heading date. (**A**) Single nucleotide polymorphisms (SNPs) in *DHD6* of 3K rice varieties. Based on these SNPs, rice varieties are divided into five haplotypes (*DHD6a–e*). Polymorphic nucleotides are shown in green boxes, with the Nipponbare sequence as reference. (**B**) Heading dates of different haplotypes of 3K rice varieties. The number of rice varieties for each haplotype is shown below each boxplot. Different letters indicate significant differences according to the one-way ANOVA and Tukey’s multiple comparisons test. (**C**) Distribution of the five haplotypes in subpopulations of rice. Aus, Aus rice varieties; Bas, Basmati; GJ, *Geng*/Japonica; XI, *Xian*/indica; Admix, accessions that cannot be clearly classified. (**D**) Geographic distribution of the five haplotypes of *DHD6*. (**E**) Model of *DHD6* and other flowering-time genes regulating flowering under long-day and short-day conditions.

Natural variation provides important insights into how genes contribute to phenotypic diversity and environmental adaptation, as well as valuable alleles for breeding. To investigate whether the natural alleles of *DHD6* affect heading date, we analyzed natural variations in coding and promoter regions of *DHD6* and heading date in 3K whole-genome-sequenced rice varieties [35,36]. *DHD6* is low in polymorphisms and only contains three single nucleotide polymorphisms (SNPs) in its coding region. Based on the three SNPs, we grouped 3K rice varieties into five haplotypes (Figure 6A). Haplotypes *DHD6a* and *DHD6b* contain the C174A variation compared with the reference genome Nipponbare (*DHD6c*) and are associated with early flowering phenotypes. In contrast, *DHD6d* and *DHD6e* haplotypes contain the C945T variation, associated with late flowering (Figure 6B). Haplotypes *DHD6a* and *DHD6b* mainly belong to Aus varieties, while *DHD6d* and *DHD6e* mainly belong to indica varieties (Figure 6C). We further analyzed the geographical distributions of different haplotypes in rice-growing regions in Africa, Asia, and Europe. The japonica allele *DHD6c* is widely distributed from low-latitude regions to high-latitude regions, whereas the indica alleles *DHD6d* and *DHD6e* are mainly distributed in low-latitude regions, including the Philippines, India, and China, and *DHD6a* and *DHD6b* are mainly distributed in low-latitude regions, including India and Bangladesh (Figure 6D). Compared to the coding region, there are more SNPs in the 2 kb promoter region. We selected five haplotypes that include most of the 3K rice varieties. Among them, hap3 and hap5 show early flowering phenotypes, and both have SNPs farther than 1 kb from the start codon ATG (Figure S4A,C). Haplotypes hap3 and hap5 mainly belong to Aus and indica varieties, respectively (Figure S4B).

## 3. Discussion

In the current study, we identified *dhd6*, an allele conferring early flowering in rice. DHD6 negatively regulates flowering time by suppressing the expression of *Ehd1*. Furthermore, by stacking the *dhd6* allele with *se14* and *phyC*, we generated a line that can complete six generations in one year. Additionally, natural alleles of *DHD6* have been identified from rice populations and are associated with varied heading dates, contributing to the regional adaptation of cultivated rice. Taken together, we propose a model in which DHD6 functions as a floral repressor by inhibiting the expression of *Ehd1* under both LD and SD conditions. Specifically, under LD conditions, DHD6 cooperates with Se14 and PHYC to reinforce this repression, thereby delaying flowering (Figure 6E).

DHD6 regulates heading date through the central regulator *Ehd1* (Figure 4). DHD6 is well conserved in plants (Figure S1), but its homolog has only been characterized in Arabidopsis (a LD plant), in which mutation of the *DHD6* homolog *APRF1*/*S2La* delays flowering by inducing the expression of *FLC* [37,38]. In contrast to the observation that mutation of *DHD6* promotes heading date in rice, overexpression of *APRF1* accelerates heading date in Arabidopsis [38]. In other words, DHD6 is conserved in the regulation of heading date in rice and Arabidopsis but has an opposite role, consistent with the differential regulations of flowering by DHD6 and APRF1 in SD and LD plants, respectively.

The double and triple mutants *dhd6 phyC*, *dhd6 se14*, and *dhd6 phyC se14* flower earlier than the single mutants (Figure 5C), indicating that these three genes function additively in regulating flowering time in rice. Se14, a demethylase, regulates the demethylation of H3K4me of *RFT1* to delay flowering [12]. PHYC regulates flowering time through Ghd7 [39]. We hypothesize that *DHD6* coordinates with *Se14* to regulate flowering time through the common target, *RFT1*, by H3K4 methylation and demethylation. It may work with PHYC likely through different yet unknown downstream targets. An additive effect is not uncommon for regulators of heading date in rice. For example, the additive effect is observed in the *ghd7 ghd8 prr37 hd1* line that flowers extremely early [40]. How exactly DHD6 regulates rice flowering needs further investigation.

The *dhd6* mutant shows an early flowering phenotype, with the seed setting rate slightly reduced. However, flowering time-related natural alleles that only have minimal negative effects on other agronomic traits can contribute to regional adaptation. For example, different natural alleles of *Pseudo-Response Regulator37* (*OsPRR37*) have been used in balancing heading date and yield [41,42]. In this study, five *DHD6* alleles were identified from the 3K rice varieties, each affecting the flowering time differentially (Figure 6). Interestingly, the three SNPs are synonymous, and the possible reason that synonymous SNPs associated with observable phenotypes is that these nucleotide changes might interfere with the mRNA expression level of the gene, which has been demonstrated in a large-scale study in yeast [43]. The exact mechanisms on how these synonymous SNPs affect *DHD6* expression need further investigation.

## 4. Materials and Methods

### 4.1. Plant Materials and Growth Conditions

Plants used in this study are of *Oryza sativa* ssp. *japonica* cv. Kitaake background, and the related mutants were identified from the fast-neutron (FN)–induced mutant population generated in KitaakeX [33,34]. The genome sequence of KitaakeX is available online (https://phytozome.jgi.doe.gov, accessed on 24 October 2017) [32]. Rice seeds were de-husked, surface-sterilized in 75% (*v*/*v*) ethanol followed by 50% (*v*/*v*) commercial bleach, and germinated on 1/2 Murashige and Skoog Medium (MS) for seven days before transferring to the greenhouse [1]. The greenhouse conditions were set as the photoperiod of light/dark (12 h/12 h) at 28 °C with a relative humidity of 80%. Heading dates of the different lines were assayed under natural long-day (LD) and short-day (SD) photoperiod conditions. For the LD conditions, plants were grown in Wuhan, China (30°28′ N), from mid-April to early October. For the SD conditions, plants were grown at Sanya, China (18°23′ N), from early December to early March. Statistical analyses of heading dates were performed using GraphPad Prism 6.0 software (GraphPad software, San Diego, CA, USA). Statistical significance was assessed using Student’s *t*-test (* *p* < 0.05, ** *p* < 0.01, *** *p* < 0.001, **** *p* < 0.0001) [44].

### 4.2. Gene Cloning and Genetic Complementation Assays

Genomic variant detection in the FN75 line was performed following the method described by Li et al. [34]. Briefly, next-generation sequence reads were mapped to the Nipponbare reference genome (version 7) using the Burrows-Wheeler Aligner–MEM algorithm (BWA version 0.7.10) with default parameters. Genomic variants, including single-base substitutions (SBSs), small insertions and deletions (<30 bp), and large structural variants (≥30 bp), were identified using SAMtools, Pindel, BreakDancer, CNVnator, and DELLY. Variants present in the nonirradiated control or detected in multiple lines were removed to retain only unique mutations. Candidate variants were manually inspected using IGV (version 2.14.1) to reduce false positives. Functional effects of mutations were annotated with SnpEff. Loss-of-function variants were defined as SBSs or Indels affecting splice sites, start or stop codons, or causing frameshifts, as well as structural variants disrupting gene regions.

The FN75 line was heterozygous in the locus controlling heading date, and the early flowering progeny of FN75 was crossed with Kitaake, and the resultant F_1_ plant self-pollinated to obtain the segregating F_2_ population. Genetic markers, including Cleaved Amplified Polymorphic Sequence (CAPS) markers with primers FN75Chr5/FR, were used in co-segregation assays of heading date with individual mutations identified from line FN75 [13].

For genetic complementation assays, the full length of the *DHD6* gene, including its native 1.6 kb promoter, was amplified from Kitaake genomic DNA using primers FN75C/F12R12. The amplicons were cloned into the pRGE vector and verified with sequencing [45]. The constructs were transformed into rice calli using the *Agrobacterium*-mediated transformation method at BioRun (Wuhan, China). The T0 plants were genotyped, and successful transformation events were identified. The homozygous T1 and T2 plants were analyzed for heading date.

### 4.3. Gene Editing

CRISPR/Cas9 technology was used to edit genes *DHD6* and *Ehd1* [46,47]. Briefly, guide RNAs (gRNAs) were designed using CRISPR-P 2.0 [48]. The pGTR plasmid was used to amplify DNA fragments (gRNA scaffolds) for GG assembly. The assembled DNA fragments were cloned into the CRISPR/Cas9 vector pRGEB32. The resultant constructs were verified with sequencing and transformed into *A. tumefaciens* for subsequent plant transformation, which was performed at BioRun (Wuhan, China). Sanger sequencing was used to genotype T0 plants, and the sequencing results were analyzed using the online program DSDecodeM [49]. Multiple independent homozygous T1 and T2 lines were assayed for heading date as described above.

### 4.4. Quantitative Real-Time PCR Assays

For the expression assay of *Ehd1*, *Hd3a*, *RFT1*, *OsMADS50*, *SIP1*, and *OsTrx1*, we harvested the top fully extended leaves from 28-day-old rice plants at 5:30 am. For the expression assay of *DHD6*, we harvested different tissues of Kitaake at the flowering stage. qRT-PCR assays were performed as previously described [50]. Briefly, total RNA of leaves was extracted with Trizol reagent (Invitrogen, Carlsbad, CA, USA). The integrity of RNA was examined using agarose gel electrophoresis (1.5%), and the concentration was measured with a NanoDrop spectrophotometer (Thermo, Waltham, MA, USA). Complementary DNA (cDNA) was reverse-transcribed using the HiScript II 1st Strand cDNA Synthesis Kit (Vazyme, Nanjing, China). SYBR Green Mix (TransGen, Beijing, China) was used for quantitative PCR, which was performed using the CFX96 Real-Time System (Bio-Rad, Hercules, CA, USA). The rice *OsActin* served as an internal control for data normalization. We used the 2 ^−∆Ct^ method to calculate the expression levels of flowering-related genes in FN75 and Kitaake. Statistical analyses were performed as described above. Primers used in qRT-PCR assays are listed in Supplemental Table S2.

### 4.5. Subcellular Localization Assays

For protein subcellular localization assays, the full length of *DHD6* was fused to the pGWB5xx-3Flag-GFP vector under the control of the *Cauliflower Mosaic Virus* (CaMV) *35S* promoter [51]. The constructs were verified with sequencing, transformed into *A. tumefaciens* strain GV3101, and infiltrated into 4-week-old *N. benthamiana* leaves with/without the nuclear localization marker. The full length of *DHD6* and *SIP1* was cloned into the pM999-YFP vector and transformed into rice protoplasts. Rice protoplast preparation and transformation were performed as described [45]. The pM999-YFP-SIP1 was used as a control. Fluorescence signals were observed under the Leica SP8 confocal scanning microscope (Leica Microsystems, Wetzlar, Germany) 18 h after transformation of rice protoplasts and 48 h after infiltration of *N. benthamiana* epidermal cells.

### 4.6. Identification of DHD6 Haplotypes

The haplotypes of *DHD6* in 3K rice accessions were retrieved from the online database RFGB (http://www.rmbreeding.cn/Index/, accessed on 1 November 2022) [52]. The japonica Nipponbare allele was used as the reference for SNP analyses. Data on heading dates and geographic distributions of 3K rice accessions were download from the International Rice Genebank (https://gringlobal.irri.org/, accessed on 5 November 2022). The map was generated using ggmap (https://github.com/dkahle/ggmap, accessed on 12 November 2022) and plotted using the ggplot2 framework (https://github.com/tidyverse/ggplot2, accessed on 12 November 2022). Statistical analyses of heading dates were performed using GraphPad Prism 6.0 software (GraphPad software, San Diego, CA, USA). Significant differences were determined according to the one-way ANOVA and Tukey’s multiple comparisons test.

## 5. Conclusions

In this study, we identified a mutated allele of *DHD6* that confers early flowering in rice and five natural alleles of *DHD6* that differentially regulate flowering time. The triple mutant *dhd6 phyC se14* has a shorter flowering time than the model rice variety Kitaake, which could be used as a fast-cycling model to accelerate functional genomics studies of rice, like the model rice line Xiaowei and the foxtail millet line Xiaomi [53,54]. Moreover, natural alleles of *DHD6* are conducive to improving the regional adaptability of cultivated rice, which may be used in rice breeding.

## Data Availability

Illumina sequencing data of FN75 are available at Sequence Read Archive (SRA) under the accession number SRR10693058. RNA-seq data are available at the National Genomics Data Center Genome Sequence Archive [55,56] under the accession number PRJCA010008. The accession numbers for genes are *DHD6* (AP014961.1), *Se14* (XM_015774997.2), *PHYC* (AF141942.1), *Ehd1* (AB477988.1), *Hd3a* (AB052942.1), and *RFT1* (AB838517.1).

## Supplementary Materials

The following supporting information can be downloaded at: https://www.mdpi.com/article/10.3390/plants14223503/s1, Figure S1: Phylogenetic analysis of DHD6 homologs from diverse organisms; Figure S2: qRT-PCR assays of genes regulating heading date; Figure S3: Subcellular localization of DHD6 in rice protoplasts; Figure S4: Natural variation in promoter region of *DHD6* is associated with the heading date; Table S1: Genes mutated in line FN75; Table S2: Primers were used in this study.
