# Supplementary material for: DHD6 Delays Flowering in Rice by Negatively Regulating the Expression of Ehd1
Source: Plants (Basel). 2025 Nov 17;14(22):3503. doi: 10.3390/plants14223503 (PMC12655961; doi:10.3390/plants14223503)
Supplement: Supplementary file 1 [file plants-14-03503-s001.zip › plants-3946811-supplementary.pdf]

DHD6 delays flowering in rice by negatively regulating the expression of *Ehd1*

Qiping Sun [1,†], Le Song [1,†], Juan Zhao [1,7], Jinxia Yun [2,8], Zhenhua Guo [1], Gan Sha [1,9], Lei Yang [1], Renjian Li [1], Rashmi Jain [4], Artur Teixeira de Araujo Jr [4], Zihao He [1], Yin Wang [1], Qun Yang [1], Jiandi Xu [3], Xia Li [2], Pamela C. Ronald [4,5,6*], and Guotian Li [1,4,5,*]

Supplemental Figures

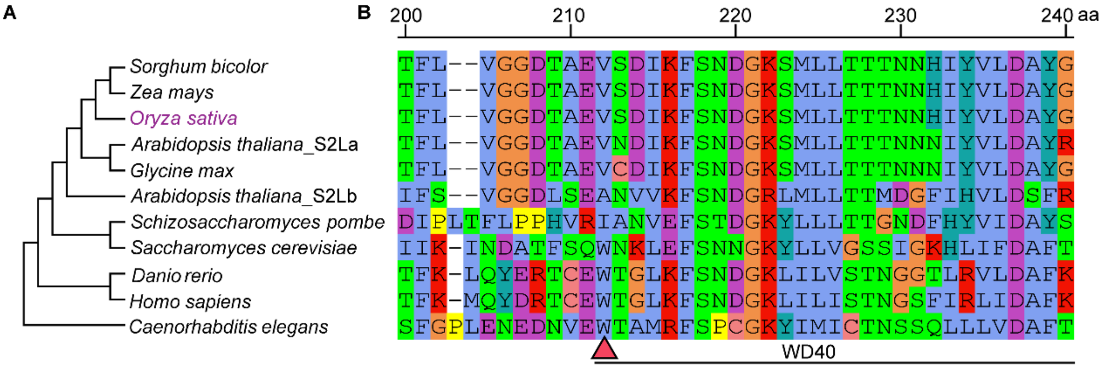

Figure S1. Phylogenetic analysis of DHD6 homologs from diverse organisms. (A) Maximum likelihood tree of full-length DHD6 proteins from representative species. DHD6 sequences from *Oryza sativa* (XP_015637863.1), *Zea mays* (NP_001152284.2), *Sorghum bicolor* (XP_021303345.1), *Glycine max* (XP_003547718.1), *Arabidopsis thaliana*_S2La (ABF85783.1), *Arabidopsis thaliana*_S2Lb (BAH19420.1), *Danio rerio* (NP_001159740.1), *Homo sapiens* (NP_079498.2), *Schizosaccharomyces pombe* (NP_595730.1), *Saccharomyces cerevisiae* (NP_012907.3), and *Caenorhabditis elegans* (NP_501280.1). The phylogenetic tree was constructed using MEGA7. The numbers indicate bootstrap values from 1000 replicates. (B) Sequence alignments of DHD6 and its homologs from different species. The black line indicates the WD40 domain. The red triangle indicates the truncated site in FN75. The sequence alignment analysis was performed using DNAMAN.

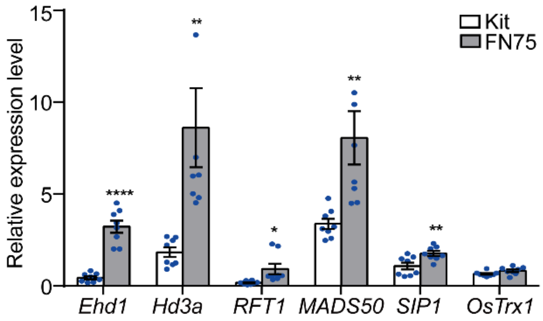

Figure S2. qRT-PCR assays of genes regulating flowering time. Total RNA was extracted from leaves of 28-day-old Kitaake and FN75 plants grown under LD conditions (n = 4). *OsActin* was used as the internal control.

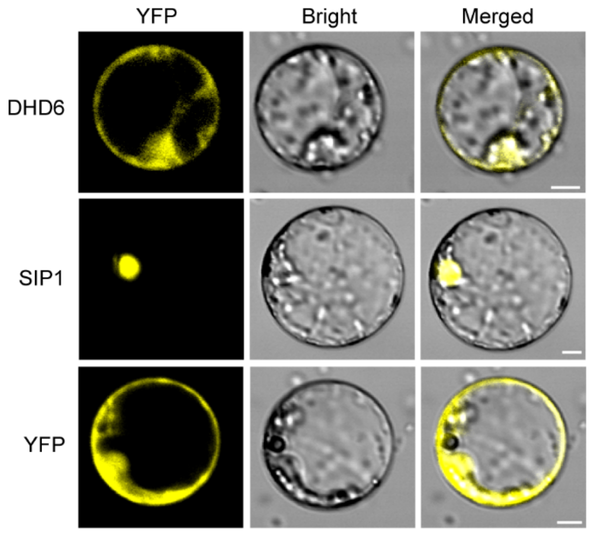

Figure S3. Subcellular localization of DHD6 in rice protoplasts. SIP1-YFP was used as a nuclear localization marker. Bars, 10 μm.

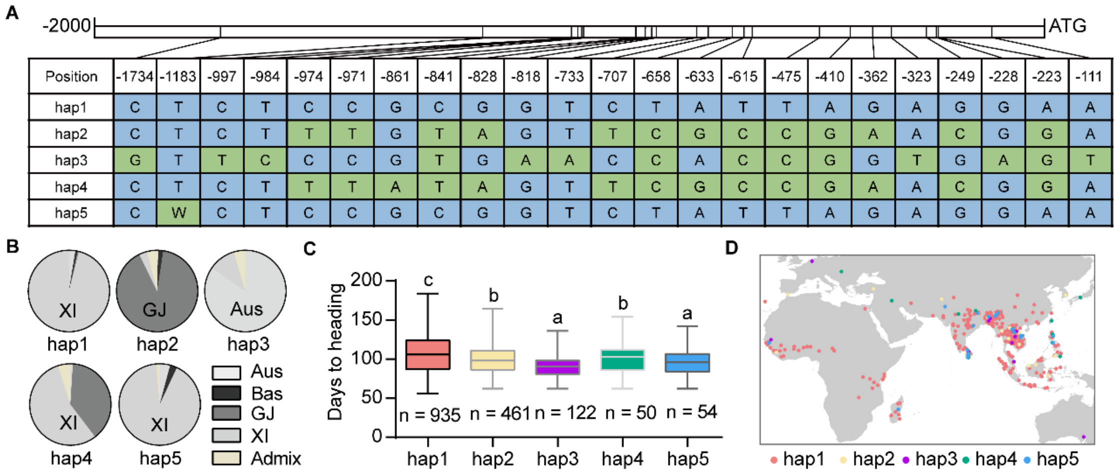

Figure S4. Natural variation in promoter region of *DHD6* is associated with the heading date. (A) Single nucleotide polymorphisms (SNPs) in *DHD6* of 1,626 rice varieties. Based on these SNPs, rice varieties are divided into five haplotypes (hap1-5). Polymorphic nucleotides are shown in green boxes with the Nipponbare sequence as the reference. (B) Distribution of the five haplotypes in subpopulations of rice. Aus, Aus rice varieties; Bas, Basmati; GJ, *Geng*/Japonica; XI, *Xian*/Indica; Admix, accessions that cannot be clearly classified. (C) Heading dates of different haplotypes of 1,626 rice varieties. The number of rice varieties for each haplotype is shown below each boxplot. Different letters indicate significant differences according to the one-way ANOVA and Tukey's multiple comparisons test. (D) Geographic distribution of the five haplotypes of *DHD6*.

Table S1. Genes mutated in line FN75.

Mutation type	Position	Gene ID	Predicted functions
Deletion	Chr12:14,310,008 – 14,314,533	LOC_Os12g24920	Transposable element protein
Deletion	Chr9:22,275,244 – 22,277,863	LOC_Os09g38768	Cell cycle control protein
Deletion	Chr9:22,348,739 – 22,351,495	LOC_Os09g38910	OsWAK92-OsWAK receptor-like protein kinase
Deletion	Chr3:22,739,267 – 22,743,374	LOC_Os03g40920	Expressed protein of unknown function
Deletion	Chr5:26,962,970 – 26,965,906	LOC_Os05g46570	WD domain, G-beta repeat domain containing protein
Deletion	Chr1:30,311,221 – 30,315,924	LOC_Os01g52710	Glycosyl transferase 8 domain containing protein
SBS	Chr9:11,671,313 – 11,676,423	LOC_Os09g19510	Senescence-induced receptor-like serine/threonine-protein kinase precursor
Inversion	Chr6: 159,330,58 – 15,922,985	LOC_Os06g28060	ATPase-like domain containing protein

Table S2. Primers used in this study.

Name	Sequence (5'-3')	Experiment
FN75Chr5/F	gtcaggttcggacaccattc	CAPS marker
FN75Chr5/R	ggccatctggagtaaaagca	
DHD6KO/F	taggtctccataccgcgccgagttttagagctagaa	CRISPR
DHD6KO/R	cgggtctcagtattcaccgactgcaccagccggg	
Ehd1KO/F5	taggtctccagaacaataacagttttagagctagaa	CRISPR
Ehd1KO/R5	cgggtctcattcttgtgatcttgcaccagccggg	
Ehd1KO/F8	taggtctccagtgaaccacctgttttagagctagaa	CRISPR
Ehd1KO/R8	cgggtctcacactgctgcaattgcaccagccggg	
FN75C/F12	caggatatccagatccagtgggatccgggcgtaaccgtacacacat	Complementation
FN75C/R12	gaacgatctactcacttagcggccgctgtgcaggaagctgtccatt	
FN75G/F3	caggatatccagatccagtgggatccatggcggcgacgctgtcgca	Overexpression
FN75G/R3	gaacgatctactcacttagcggccgcctaattcgaactggaattgt	
OsActin-RT-F	caggccgtcctctctctgta	qRT-PCR
OsActin-RT-R	aaggatagcatgggggagag	
OsMADS50-RT-F	aaagctgacgctgatggtttg	qRT-PCR
OsMADS50-RT-R	gtttcgacatccatgttgtc	
Ehd1-RT-F	gttgccagtcatctgcagaa	qRT-PCR
Ehd1-RT-R	ggatgtggatcatgagacat	
Hd3a-RT-F	agcccaagtgaccctaacct	qRT-PCR
Hd3a-RT-R	gttgtagagctcggcgaagt	
RFT1-RT-F	tgggttagctgacctagattcaaa	qRT-PCR
RFT1-RT-R	gccaaccacaagaggatcgt	
SIP1-RT-F	catcgaaggtagccaaagtg	qRT-PCR
SIP1-RT-R	aatggattcagtgcagggtt	
OsTrx1-RT-F	ctttcgtgggtatagagctgttcat	qRT-PCR
OsTrx1-RT-R	accatcatacggcacttatcacatt	
DHD6-RT-F	cttcgtgtaaatgcttgtcagggta	qRT-PCR
DHD6-RT-R	accaaccaagaaagtgtcaaaggga	
DHD6/GFP-F	acgcgtcgacatggcggcgacgct	GFP
DHD6/GFP-R	ccggaattcattcgaactggaattgttgg	
DHD6-YFP-F	ctagactcgagatggcggcgacgct	YFP
DHD6-YFP-R	tccatcccgggagcggtaccattcgaactggaattgttgg	
SIP1-YFP-F	cgggggactctagactcgagatggggaagaagaagaag	YFP
SIP1-YFP-R	tccatcccgggagcggtacccacaatattccagttcagag	

Notes: CAPS, Cleaved Amplified Polymorphic Sequence; CRISPR, Clustered Regularly Interspaced Short Palindromic Repeats; qRT-PCR, Quantitative Real-time PCR; GFP, Green fluorescent protein; YFP, Yellow Fluorescent Protein.
